# Effect of red blood cell processing on hemoglobin increment in oncology patients

**DOI:** 10.1016/j.htct.2026.106503

**Published:** 2026-09-05

**Authors:** Kshitija Mittal, Manuru Sameer, Ravneet Kaur, Awadhesh Kumar Pandey, Paramjit Kaur, Gagandeep Kaur, Shivangi Sharma

**Affiliations:** a Department of Transfusion Medicine, Government Medical College and Hospital, Chandigarh, India; b Department of Radiation and Oncology, Government Medical College and Hospital, Chandigarh, India; c Government Medical College and Hospital, Chandigarh, India

**Keywords:** Red blood cells, Component processing, Hemoglobin increment, Oncology patients

## Abstract

**Objective:**

This study aimed to evaluate the effect of red blood cell processing on the hemoglobin increment in oncology patients requiring packed red blood cell transfusions.

**Material and methods:**

The study population in this prospective, open-label, randomized controlled trial included healthy blood donors and oncology patients. Only first-time male donors aged 18 to 30 years with hemoglobin levels between 12.5 and 15 g/dL who were donating whole blood were included. Whole blood was collected in 450 mL triple or quintuple blood bag systems with integral leukoreduction filters. The triple bags were processed using the platelet-rich plasma method (non-Saline-Adenine-Glucose-Mannitol), whereas the quintuple bags were processed via the buffy coat method and leukoreduced using inline leukofilters (leukoreduced Saline-Adenine-Glucose-Mannitol). Enrolled patients were randomly allocated to two groups: Group I received non- Saline-Adenine-Glucose-Mannitol packed red blood units, and Group II received leukoreduced Saline-Adenine-Glucose-Mannitol packed red blood units.

**Results:**

Group II had red blood cell volume loss of 33.81 ± 3.49 mL during processing (p-value < 0.001). The calculated total hemoglobin content was significantly higher in Group I (67.75 ± 3.18 versus 62.47 ± 3.62 g/unit; p value < 0.001). No significant difference was observed between actual total hemoglobin content of Group I and Group II (47.51 ± 2.02 versus 46.44 ± 5.66 g/unit, p = 0.119). No significant difference was observed in the mean hemoglobin increment (Group I: 1.85 ± 0.91 g/dL versus Group II: 1.83 ± 0.64 g/dL, p = 0.887).

**Conclusion:**

Loss of hemoglobin during processing did not affect the hemoglobin increment in oncology patients in our study.

## Introduction

Blood transfusion services are the cornerstone of the modern health care system [1]. Packed red blood cells (PRBCs) are the most commonly transfused blood component [2]. The quality of the PRBC unit is critical for achieving optimal clinical outcomes following transfusion. Studies have shown that PRBC quality may be affected by blood donor characteristics, patient characteristics, and the whole blood processing method [3].

PRBCs can be prepared from whole blood using two processing methods: platelet rich plasma (PRP) and buffy coat (BC) [4]. No red blood cell loss is observed during the processing of whole blood to PRBCs via the PRP method without Saline-Adenine-Glucose-Mannitol (non-SAGM). Conversely, during the preparation of leukoreduced Saline-Adenine-Glucose-Mannitol (SAGM) PRBCs via the BC method and inline leukofiltration, red blood cell loss occurs at two distinct stages: initially during BC removal, which results in a loss of 10–20 mL of RBCs, and subsequently during leukofiltration, which accounts for a further loss of 20–30 mL, culminating in a total volume loss of 30–50 mL [4., 5., 6., 7.]. Leukoreduced SAGM PRBC units exhibit a significantly lower total hemoglobin (THb) content (51 g) compared to non-SAGM PRBC units (58 g) [6].

The hemoglobin (Hb) increment following the transfusion of each PRBC unit is a critical metric of transfusion efficacy. One unit of PRBC should raise the Hb of an adult by 1 g/dL, however, this is not true under all conditions [6]. Studies have shown a statistically significant correlation between the Hb increment in patients with the total Hb dose given [8].

Studies in the past have reported variations in Hb content in PRBC units prepared by different processing methods. However, the literature regarding the impact of red blood cell processing on Hb increment in patients is scant. Thus, this study aimed to evaluate the effect of red blood cell processing on the hemoglobin increment in oncology patients undergoing radiotherapy or chemotherapy requiring PRBC transfusion.

## Materials and methods

The study was conducted in the Department of Transfusion Medicine of a tertiary care hospital of North India from 1st January 2023 to 31st December 2023 after obtaining the necessary approval from the Institute Ethics Committee (No. GMCH/IEC/836R/2022/236 dated 09.12.2022). The study was a prospective open label randomized controlled trial registered in the Clinical Trials Registry of India (CTRI/ 2023/ 03/ 050955). All healthy blood donors donating whole blood were screened according to the criteria laid down by the National Regulatory Authority [9]. Male donors aged 18–30 years with pre-donation Hb levels between 12.5 and 15 g/dL who were donating whole blood for the first time, and whose blood was collected in triple or quintuple blood bags with inline leukoreduction filters, were considered for the study. Donors were excluded if they failed to meet eligibility criteria, demonstrated reactivity for any transfusion-transmitted infection (TTI), or had volume deviations. The donors were informed about the nature of the study, and prior written informed consent was obtained.

### Methodology

For pre-donation Hb estimation, a 2 mL sample of blood was collected in ethylenediaminetetraacetic acid (EDTA) tubes from all eligible blood donors, and donor demographics were recorded using a standardized proforma. Hb levels were determined using a fully automated hematology cell counter (Erba H360, Transasia Biomedical, India).

### Whole blood collection

Whole blood was collected from 156 healthy male blood donors either in 450 mL triple (Terumo Penpol, Thiruvananthapuram, India) blood bags with Citrate-Phosphate-Dextrose-Adenine (CPDA) anticoagulant (n = 78) or quintuple blood bags with inline leucofilter (Terumo Penpol, Thiruvananthapuram, India) with Citrate-Phosphate-Dextrose (CPD) anticoagulant and SAGM additive solution (n = 78) as per the departmental standard operating procedure (SOP).

### Blood transportation and processing

Blood bags were transported under controlled conditions at the optimum prescribed temperature to the component preparation laboratory and were processed as per the departmental SOP. The cryofuge settings were maintained constant, and component preparation was performed using the same refrigerated centrifuge (Heraeus, Thermo Scientific, Germany) throughout the study period.

The triple blood bags were processed using the PRP method. The centrifugation parameters consisted of a relative centrifugal force of 468 × g (acceleration profile: 8; deceleration profile: 1) at 22 °C for 10 min. The supernatant PRP was transferred to a platelet storage satellite bag using a plasma expressor (Terumo Penpol, Thiruvananthapuram, India) while the settled PRBCs remained in the primary bag. The non-SAGM PRBC unit was labeled and stored in a blood storage refrigerator at 2–6 °C.

The quintuple blood bags with inline leukofilter were processed using the buffy coat (BC) method and leukoreduced using inline leukofilters on the same day. The centrifugation parameters consisted of a relative centrifugal force of 4822 × g (acceleration profile: 9; deceleration profile: 5) at 22 °C for 10 min. Whole blood units were separated using an automated component extractor (TACE-II, Terumo Penpol, Thiruvananthapuram, India). The separated BC-depleted PRBCs were transferred into a satellite bag. The SAGM additive solution from an attached satellite bag was passed through the inline leukofilter into the satellite bag containing the BC-depleted PRBCs and the PRBCs and SAGM were thoroughly mixed. The pre-filtration weight of the SAGM PRBC unit was measured using a double pan weighing scale (Imperial Biotech, India). The SAGM PRBC unit was then subjected to inline leukofiltration. The post-filtration weight of the SAGM PRBC unit was also measured and the volume lost during leukofiltration was calculated. The leukoreduced SAGM PRBC unit was labelled and stored in a blood storage refrigerator at 2–6 °C.

### Sampling

A sample was taken from each PRBC unit to measure the actual THb content and hematocrit using a fully automated hematology analyzer (Erba H360, Transasia Bio-Medicals, India) after stripping the integral tubing segment thrice. Another sample was taken on the following day after component preparation. All PRBC units were shifted to the inventory for issue to the study patients.

### Calculations

**Figure 1:**
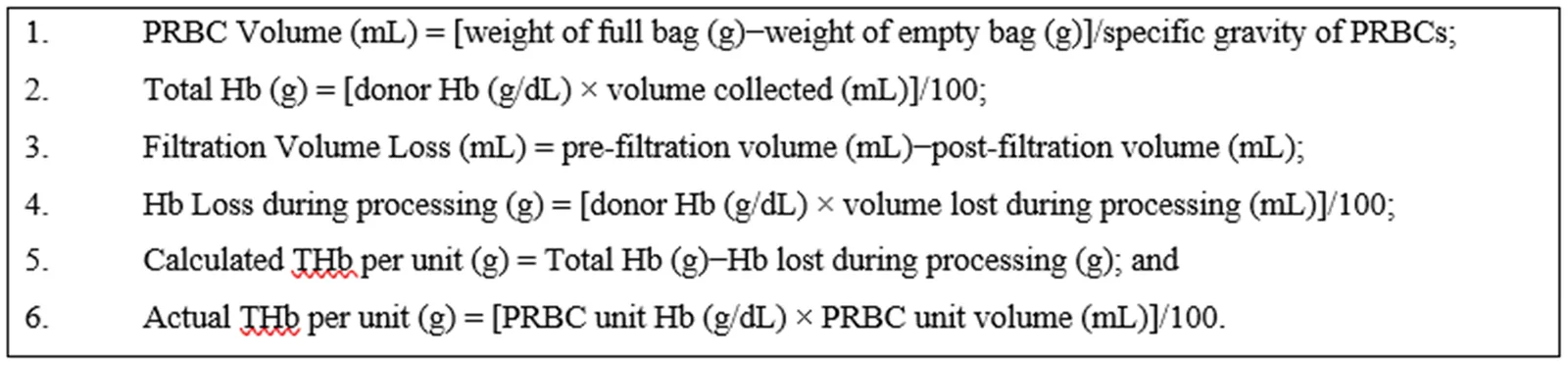
Mathematical formulas for blood component quality control parameters. Equations 1 through 6 represent the standardized calculations used to evaluate PRBC unit physical characteristics, processing losses, and final hemoglobin content. Hb: hemoglobin; PRBC: packed red blood cells; THb: total hemoglobin content.

Mathematical equations utilized for calculating packed red blood cell (PRBC) volumes, hemoglobin recovery, filtration losses, and total hemoglobin content are detailed in Figure 1.

### Patient enrollment

Oncology patients on radiotherapy or chemotherapy who received treatment at GMCH and required PRBC transfusions were included in the study. Patients who received treatment outside GMCH, patients suffering from esophageal cancer, head and neck cancer, very advanced cases, and patients with active bleeding were excluded from the study. A total of 156 patients were enrolled in the study.

### Randomization and group allocation

Oncology patients were randomized into two equal groups via computer-generated random numbers, with allocation concealment maintained by serially numbered opaque sealed envelopes. Group I (n = 78) received non-SAGM PRBC units, and Group II (n = 78) received leukoreduced SAGM PRBC units. Baseline demographics and histories were documented via a pre-structured case report form, and the body mass index (BMI) was calculated as weight (kg)/height squared (m^2^).

### Transfusion of packed red blood cells

Only one PRBC unit was transfused at a time to the patient. The same patient was enrolled again only after a gap of two weeks. Two mL EDTA samples were drawn before each transfusion and within 24 h after the PRBC unit transfusion for pre- and post-transfusion Hb estimation which was done using a fully automated hematology cell counter (Erba H360, Transasia Biomedical, India).

### Outcome measures

The Primary outcome was Hb increment in both the study arms and the secondary outcome was untoward adverse transfusion reactions.

## Statistical analysis

Categorical data were presented as counts and percentages. Continuous variables were expressed as means ± standard deviation. The normality of quantitative data was assessed using the Kolmogorov–Smirnov test. For normally distributed data, an independent samples t-test was applied to compare the two groups. Categorical variables were compared using the Pearson chi-square test or Fisher's exact test, as appropriate. All statistical tests were two-sided, and a p-value <0.05 was considered statistically significant. Statistical analyses were conducted using IBM SPSS Statistics for Windows, version 22.0 (IBM Corp., Armonk, NY, USA).

### Sample size and its basis

According to the National Regulatory Authority guidelines, only 1% of the prepared PRBCs and components are evaluated for quality control [9]. Given an annual blood collection of 15,000 units, quality control is performed on approximately 150 units per year. With these 150 units serving as the finite target population, the required sample size was calculated to be 109 individuals using a 95% confidence level and a 5% margin of error. To account for potential attrition, an additional 20% was added, resulting in a final calculated sample size of 130 subjects.

## Results

**Figure 2:**
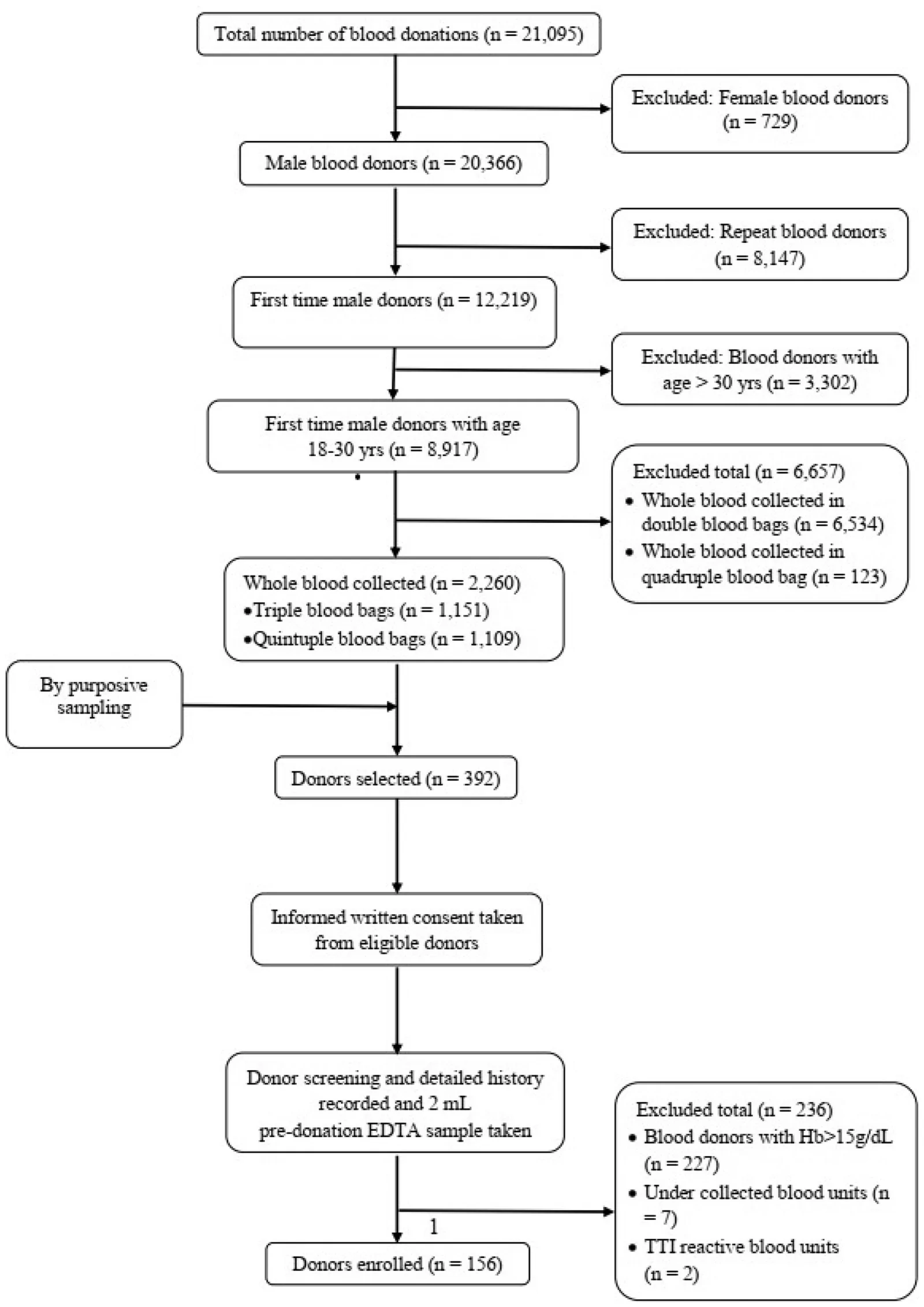
Consort diagram of the whole blood donors and blood component processing.

Throughout the study period, 21,095 individuals donated whole blood. Of these, 18,835 blood donors were excluded from the study as per the inclusion and exclusion criteria. Of the remaining 2260 blood donors where whole blood was collected in triple and quintuple blood bags with integral leukofilter, 392 blood donors were selected using the purposive sampling method. Out of these, 227 blood donors were excluded due to pre-donation Hb >15 g/dL, seven units were under collected and two units were TTI positive. Thus, a total of 156 blood donors were finally included in the study (Figure 2).

**Figure 3:**
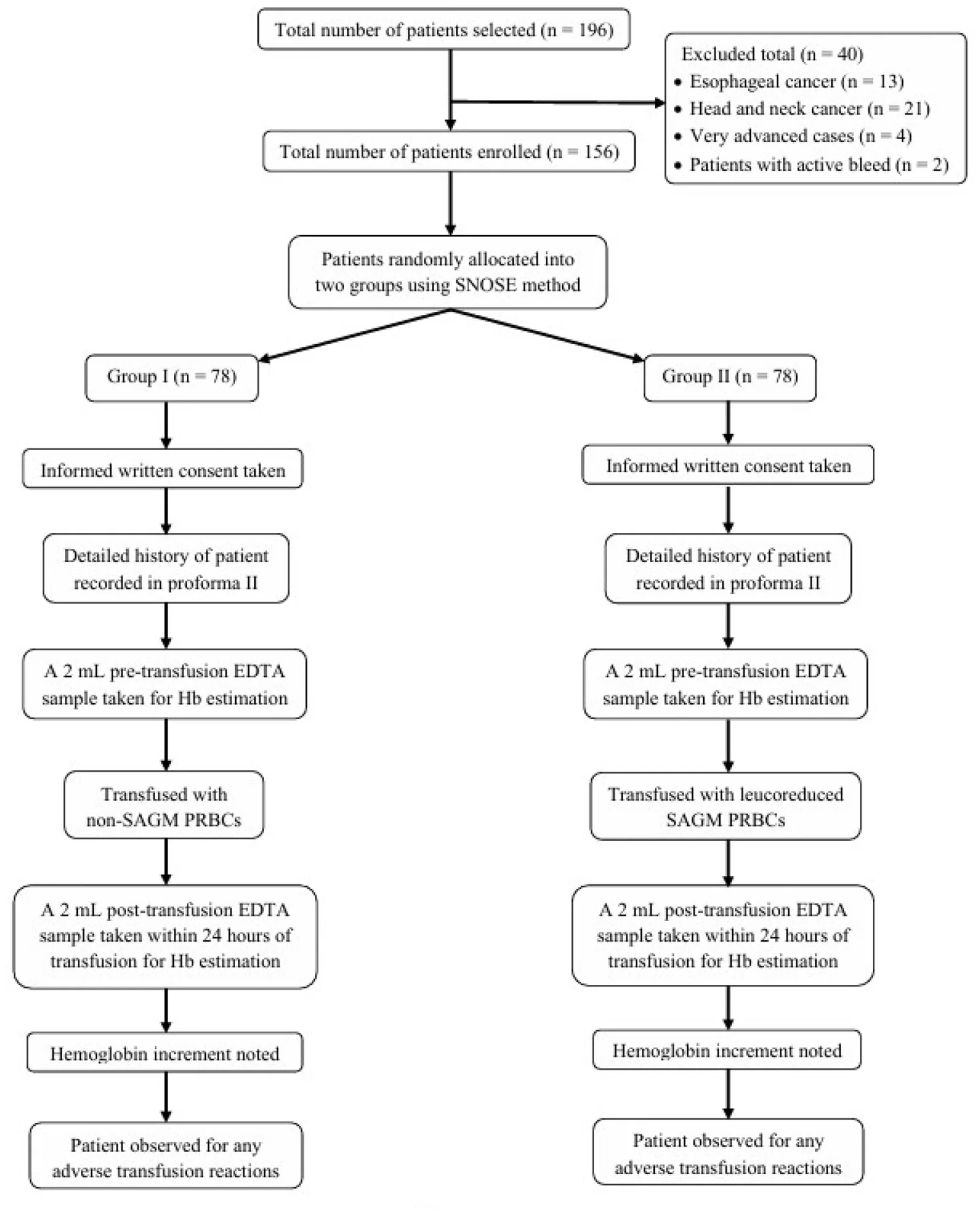
Consort diagram of patients.

Out of 196 oncology patients considered, 40 patients were excluded as 13 patients were diagnosed with esophageal cancer, 21 patients with head and neck cancer, four patients were in a very advanced stage of cancer and two patients were actively bleeding. Hence, 156 patients (males n = 58; females n = 98) were enrolled in the study. The enrolled patients were mainly diagnosed with breast, lung, gastrointestinal, genitourinary, bone, hematological, secondary metastasis and thyroid malignancies. The study cohort comprised 156 patients divided equally into two transfusion arms (Figure 3). Group I (25 males: 32%; 53 females: 68%) received non-SAGM PRBC units, while Group II (33 males: 42%; 45 females: 58%) received leukoreduced SAGM PRBC units.

### Blood donor characteristics

#### Age and pre-donation hemoglobin

The mean age of donors from whom whole blood was collected in 450 mL triple blood bags and quintuple blood bags with inline leukofilter was 24.47 ± 3.81 years and 23.76 ± 3.79 years, respectively. The mean age difference between the blood donor groups was found to be statistically insignificant (p = 0.239 - paired sample t-test).

The mean pre-donation Hb of donors from whom blood was collected in 450 mL triple blood bags and quintuple blood bags with inline leukofilter was 14.66 ± 0.49 g/dL and 14.54 ± 0.66 g/dL, respectively. No significant difference was observed in mean pre-donation Hb of donors between the groups (p = 0.198 - paired sample t-test).

### Blood bag characteristics

#### Volume of whole blood units and total hemoglobin

The mean volume of whole blood collected in triple blood bags was 462.74 ± 13.3 mL while that collected in quintuple blood bags with inline leukofilter was 463.28 ± 14.66 mL. The mean volume of whole blood collected was statistically similar in both groups (p = 0.810 - paired sample t-test).

No significant difference was observed in the mean total Hb collected in triple blood bags and quintuple blood bags with inline leukofilter (67.75 ± 3.18 g vs. 67.38 ± 4.16 g, respectively; p = 0.530 - paired sample t-test).

### Packed red blood cell unit parameters

#### Volume of packed red blood cell units and red blood cell volume loss during processing

A significant difference was observed in mean volume of PRBC units between the groups (p = 0.000). No red blood cell volume loss was observed in the preparation of non-SAGM PRBC units while leukoreduced SAGM PRBC units had a red blood cell volume loss of 33.81 ± 3.49 mL during processing (p < 0.001; Table 1).

**Table 1:** Packed red blood cell characteristics.

PRBC characteristic	Non-SAGM PRBC units	Leukoreduced SAGM PRBC units	*p-value
	Mean ± SD	Mean ± SD	
Volume (mL)	268.15 ± 5.21	235.68 ± 16.66	0.000
Red blood cell volume lost during processing (mL)	0.00 ± 0.00	33.81 ± 3.49	0.000
Hematocrit (%)	66.33 ± 0.81	57.41 ± 1.83	0.000
Mathematically calculated total Hb content per PRBC unit (g/unit)	67.75 ± 3.18	62.47 ± 3.62	0.000
Actual total Hb content per PRBC unit (g/unit)	47.51 ± 2.02	46.44 ± 5.66	0.119

⁎ p-value calculated using paired sample t-test SAGM: Saline-Adenine-Glucose-Mannitol; PRBC: Packed red blood cell; Hb: hemoglobin.

### Hematocrit

The mean hematocrit of the non-SAGM PRBC units was significantly higher compared to leukoreduced SAGM PRBC units (p < 0.001; Table 1).

### Hemoglobin lost during processing

The total Hb lost during processing using the BC method with the inline leukofiltration was found to be statistically significant compared to the PRP method (p < 0.001; Table 1)

### Mathematically calculated total hemoglobin content and actual total hemoglobin content per packed red blood cell unit

The mathematically calculated THb content was significantly higher in non-SAGM PRBC units compared to the leukoreduced SAGM PRBC units (p < 0.001). However, no significant difference was observed between the actual THb content of non-SAGM PRBC units and leukoreduced SAGM PRBC units (p = 0.119; Table 1).

### Patient characteristics

#### Age and body mass index

The mean ages of patients in Group I and Group II were statistically similar (p = 0.820; range: 19–77 years). No statistically significant difference was found in mean BMI of the patients between the study arms (p = 0.457; Table 2).

**Table 2:** Patient characteristics.

Patient characteristic	Group I	Group II	*p-value
	Mean ± SD	Mean ± SD	
Age (years)	55.44 ± 10.89	55.88 ± 13.56	0.820
Weight (kg)	51.68 ± 11.15	53.22 ± 11.51	
Height (m)	1.57 ± 0.09	1.57 ± 0.10	
BMI (kg/m^2^)	21.16 ± 4.71	21.74 ± 5.04	0.457

⁎ p-value calculated using paired sample t-test SD: Standard Deviation; BMI: Body mass index.

### Transfusion parameters

#### Age of the packed red blood cell units at the time of transfusion

The ages of the PRBC units at the time of transfusion in Group I and Group II patients were comparable (15.29 ± 7.64 days vs. 16.71 ± 9.73 days; p = 0.316 - paired sample t-test).

#### Post-transfusion hemoglobin estimation time

No significant difference was observed in the estimated post-transfusion Hb time between Group I and Group II patients (12.49 ± 1.67 h vs. 12.86 ± 1.32 h, respectively; p = 0.125 - paired sample t-test).

### Outcome parameters

#### Pre- and post-transfusion hemoglobin levels of patients

The mean pre-transfusion Hb levels of Group I and Group II patients were comparable (7.73 ± 1.17 g/dL vs. 7.59 ± 1.17 g/dL, respectively; p = 0.460; Table 3). The mean post-transfusion Hb levels of Group I and Group II patients were also comparable with no significant difference (9.58 ± 1.48 g/dL vs. 9.43 ± 1.11 g/dL, respectively; p = 0.456; Table 3).

**Table 3:** Patient outcome parameters.

Patient outcome parameter	Group I	Group II	*p-value
	Mean ± SD	Mean ± SD	
Pre-transfusion Hb (g/dL)	7.73 ± 1.17	7.59 ± 1.17	0.460
Post-transfusion Hb (g/dL)	9.58 ± 1.48 g/dL	9.43 ± 1.11	0.456
Hb increment (g/dL)	1.85 ± 0.91	1.83 ± 0.64	0.887

⁎ p-value calculated using paired sample t-test SD: Standard Deviation; Hb: Hemoglobin.

### Hemoglobin increment

No significant difference was observed in the mean Hb increment in both study arms (Group I: 1.85 ± 0.91 g/dL vs. Group II: 1.83 ± 0.64 g/dL; p = 0.887; Table 3).

### Adverse transfusion reactions

No untoward adverse transfusion reactions were observed in either of the study groups.

## Discussion

PRBCs are one of the major blood components transfused in the medical and surgical practice [10]. PRBC units differ with respect to their Hb content, and volume despite uniform collection [11] with this difference being able to affect Hb increment when transfused to patients. Thus, this study was conducted to determine the effect of whole blood processing on Hb increment in oncology patients requiring PRBC transfusions.

To minimize variability among the collected blood units, this study included only first-time male blood donors aged 18–30 years with pre-donation Hb levels of 12.5–15.0 g/dL. Whole blood (450 ± 10% mL) was collected either in triple or quintuple blood bags with an inline leukofilter. The mean total Hb collected in both the blood bags was comparable (p = 0.530) which could be due to comparable pre-donation Hb of the blood donors (p = 0.198) and the volume of whole blood collected (p = 0.810).

The mean PRBC volume was significantly higher in the non-SAGM PRBC units processed using the PRP method compared to leukoreduced SAGM PRBC units processed using the BC method and leukoreduced using an inline leukofilter (p < 0.001). A mean volume loss of 33.81 ± 3.49 mL (p < 0.001) was noted during the processing of quintuple blood bags with an inline leukofilter. This loss in volume could be attributed to removal of BC and leukofiltration of PRBC units. A prospective cross-sectional study conducted at our center on 100 whole blood units collected in 450 mL quintuple blood bags with an inline leukofilter also reported a volume loss of 29.9 ± 15.8 mL during BC processing and leukofiltration [12]. Similarly, another blood center reported a volume loss of 35 ± 2.3 mL when 50 whole blood units were subjected to leukofiltration using inline leukofilters [13].

An Hb loss of 4.91 ± 0.62 g/unit was seen in quintuple blood bags with an inline leukofilter. A prospective study from the region also reported a mean Hb loss of 3.34 ± 1.75 g/unit during removal of BC while processing 450 mL whole blood units collected in quadruple blood bags [14]. In another study, the authors observed Hb loss of 2 g/unit during leukofiltration of PRBCs using laboratory-side red blood cell leukofilters [7].

The actual THb content per PRBC unit is the most important parameter for the quality of the product [13]. Rudrappan et al. found a significant difference between actual THb content of PRBC units prepared using the PRP method and those prepared using the BC method (58 g vs. 51 g respectively; p < 0.0001) [6]. However, in the present study, the actual THb content of non-SAGM PRBC units and leukoreduced SAGM PRBC units was comparable with no significant difference (47.51 ± 2.02 g vs. 46.44 ± 5.66 g; p = 0.119). Agnihotri et al. observed the actual THb content of leukoreduced SAGM PRBC units prepared from 450 mL triple blood bags with an inline leukofilter and 350 mL double blood bags filtered using leucocyte reduction filters ranged from 42.3–80.8 g (mean: 61.3 ± 6.9 g) [13]. Jain et al. noted the actual THb content of PRBC units prepared from 350 mL double blood bags, 450 mL triple blood bags and 450 mL quadruple blood bags ranged from 30.77–87.36 g (mean: 52.91 ± 9.99 g/unit) [14]. The lower mean value of actual THb content of PRBC units in the present study could be due to lower pre-donation Hb of blood donors (12.5–15.0 g/dL) compared to other studies where the pre-donation Hb was up to 18.0 g/dL.

This study evaluated the effect of red blood cell processing on 156 oncology patients who were randomized to Group I or Group II. Patients suffering from esophageal carcinoma, head and neck carcinoma, very advanced stages of carcinoma and actively bleeding were not included in the study as these patients are very sick and may require multiple units of PRBCs within 24 h.

In this study, the mean ages of the PRBC units at the time of transfusion in Group I and Group II patients were similar (p = 0.316). Previous studies have reported variable effects of PRBC storage duration on post-transfusion Hb increments in recipients [15,16]. Recipient factors such as age, weight, and BMI have also been found to differentially impact Hb increment after PRBC transfusion [15,17,18]. In the current study, no statistically significant difference was found in mean BMI of the patients between the study groups (p = 0.457).

Patient’s pre-transfusion Hb is an important determinant in predicting Hb increment. In one study, an inverse relation was found between pre-transfusion Hb levels and Hb increment with the highest increment in patients having a baseline Hb <7 g/dL and lowest in patients having a baseline Hb >8 g/dL (1.26 ± 0.95 g/dL vs. 0.96 ± 0.89 g/dL) [15]. However, in the present study, the mean pre-transfusion Hb values of Group I and Group II patients were comparable (p = 0.460).

The timing of post-transfusion testing is also a critical determinant of the recorded Hb increment. This study stipulated that post-transfusion Hb levels be measured within 24 h, as previous data demonstrate no significant difference in Hb equilibration at 1, 4, and 24 h following a PRBC transfusion [19]. In this study the post-transfusion Hb was comparable between both groups (p = 0.125).

Hemoglobin increment after each PRBC unit transfusion is one of the ways to measure transfusion efficacy. The expected Hb increment following one unit of PRBC transfusion is 1 g/dL [6]. In the current study, PRBC transfusions were effective, as the mean Hb increment was greater than 1 g/dL in both study groups. Similarly, Reikvam et al. observed a statistically significant correlation between Hb increment and the total Hb dose administered (p = 0.0024; r = 0.4120) [8]. In the present cohort, the mean Hb increment in Group I and Group II patients was statistically similar, which could be attributed to the equivalent actual THb content of the transfused PRBC units (p = 0.119). Similarly, Saqlain et al. found no significant difference in mean Hb increment between leukodepleted and non-leukodepleted PRBC units (2.44 ± 0.46 g/dL vs. 2.40 ± 0.41 g/dL; p = 0.650) [20].

## Conclusion

To conclude, processing of leukoreduced SAGM PRBC units from quintuple blood bags using the BC method and leukoreduction using an inline leukofilter leads to a loss of volume which further leads to Hb loss. However, the loss of Hb during processing did not affect the Hb increment in oncology patients in this study. Patient's underlying condition could be an important factor affecting the transfusion outcome.

## Data availability

The data that support the findings of this study are available from the corresponding author upon reasonable request.

## Conflicts of interest

The authors declare no conflicts of interest.
